# What is the impact of acquired immunity on the transmission of schistosomiasis and the efficacy of current and planned mass drug administration programmes?

**DOI:** 10.1371/journal.pntd.0009946

**Published:** 2021-12-01

**Authors:** Klodeta Kura, Robert J. Hardwick, James E. Truscott, Roy M. Anderson

**Affiliations:** 1 London Centre for Neglected Tropical Disease Research, London, United Kingdom; 2 Department of Infectious Disease Epidemiology, School of Public Health, Faculty of Medicine, St Mary’s Campus, Imperial College London, London, United Kingdom; 3 MRC Centre for Global Infectious Disease Analysis, London, United Kingdom; 4 The DeWorm3 Project, The Natural History Museum of London, London, United Kingdom; University of Glasgow School of Life Sciences, UNITED KINGDOM

## Abstract

Schistosomiasis causes severe morbidity in many countries with endemic infection with the schistosome digenean parasites in Africa and Asia. To control and eliminate the disease resulting from infection, regular mass drug administration (MDA) is used, with a focus on school-aged children (SAC; 5–14 years of age). In some high transmission settings, the World Health Organization (WHO) also recommends the inclusion of at-risk adults in MDA treatment programmes. The question of whether ecology (age-dependant exposure) or immunity (resistance to reinfection), or some combination of both, determines the form of observed convex age-intensity profile is still unresolved, but there is a growing body of evidence that the human hosts acquire some partial level of immunity after a long period of repeated exposure to infection. In the majority of past research modelling schistosome transmission and the impact of MDA programmes, the effect of acquired immunity has not been taken into account. Past work has been based on the assumption that age-related contact rates generate convex horizontal age-intensity profiles. In this paper, we use an individual based stochastic model of transmission and MDA impact to explore the effect of acquired immunity in defined MDA programmes. Compared with scenarios with no immunity, we find that acquired immunity makes the MDA programme less effective with a slower decrease in the prevalence of infection. Therefore, the time to achieve morbidity control and elimination as a public health problem is longer than predicted by models with just age-related exposure and no build-up of immunity. The level of impact depends on the baseline prevalence prior to treatment (the magnitude of the basic reproductive number R_0_) and the treatment frequency, among other factors. We find that immunity has a larger impact within moderate to high transmission settings such that it is very unlikely to achieve morbidity and transmission control employing current MDA programmes.

## Author summary

The question of ‘ecology or immunology’ as determinants of the shape of age intensity profiles of infection is still not resolved, but a degree of immunity is believed to slowly build up over long periods of exposure. In this study we show that acquired immunity dependent on the integral of past exposure to infection has a strong effect on the potential impact of MDA programmes to control morbidity induced by schistosome infection. We employ an individual based stochastic model of parasite transmission and control to demonstrate the effects of acquired immunity. In particular, we show that MDA programmes are less effective if human hosts acquire some level of immunity in proportion to the sum of an individual’s past exposure to infection. If acquired immunity is acting with strength, in some moderate to high transmission settings, we show that the current treatment guidelines are unlikely to achieve morbidity control and elimination as a public health problem. This suggests that programme adaptations to increase the frequency of treatment where at all possible are required. This study aims to provide health workers with quantitative tools to assess what treatment strategies are best applied in given epidemiological settings.

## Introduction

Schistosomiasis remains an endemic parasitic disease in many regions of the world and inflicts significant levels of human morbidity [1,2]. An individual becomes infected when the cercarial larval forms of the parasite (released by the intermediate host which are various species of freshwater snails), penetrate the skin during contact with contaminated water [3]. The disease caused by the parasite is an intestinal or urogenital disease and the main trematode species known to infect humans in Africa and Asia are *S*. *mansoni*, *S*. *haematobium* and *S*. *japonicum*.

Mass drug administration (MDA), using the drug praziquantel (PZQ), is the main form of control at present, alongside behaviour modification and improvement in sanitation. Current work on potential vaccine candidates is promising, but at an early stage at present [4–7].

The World Health Organization (WHO) has set guidelines to control the morbidity induced by infection and these have recently been reviewed in the new NTD control Roadmap for 2021-to 2030 [1,8]. The first aim relates to the morbidity control, which is defined as lowering the prevalence of the heavy intensity infections (>50 eggs per 10 ml for *S*.*haematobium*) in school age children (SAC; 5–14 years of age) to less than 5%. The second aim is elimination as a public health problem (EPHP), defined as lowering the prevalence of the heavy intensity infections in SAC to less than 1%. The current recommended treatment frequencies depend on the baseline prevalence among SAC (prior to MDA initiation) which reflect the magnitude of the basic reproductive number R_0_ [9]. In low transmission settings (<10% baseline prevalence among SAC) it is recommended that 75% of SAC should be treated every three years. In moderate transmission settings (10−50% baseline prevalence among SAC) it is recommended that 75% of SAC should be treated once every two years while in high transmission settings (≥50% baseline prevalence among SAC) treatment should be given annually. Recent work from the Bill and Melinda Gates Foundation SCORE project has also examined the benefits of twice annual treatment of SAC and Adults in different arms of a field based set of trials [10].

In most countries current MDA programmes are mostly focused on treating SAC only, but inclusion of adults at risk particularly in high transmission settings is also recommended [11]. Past modelling work on *S*.*haematobium* has shown that the morbidity control goal can be achieved by using the WHO-recommended treatment guidelines, but EPHP goal can only be achieved in low to some moderate transmission settings [12,13]. In some moderate to high prevalence settings, community wide treatment is required to achieve the WHO recommended targets.

**Fig 1:**
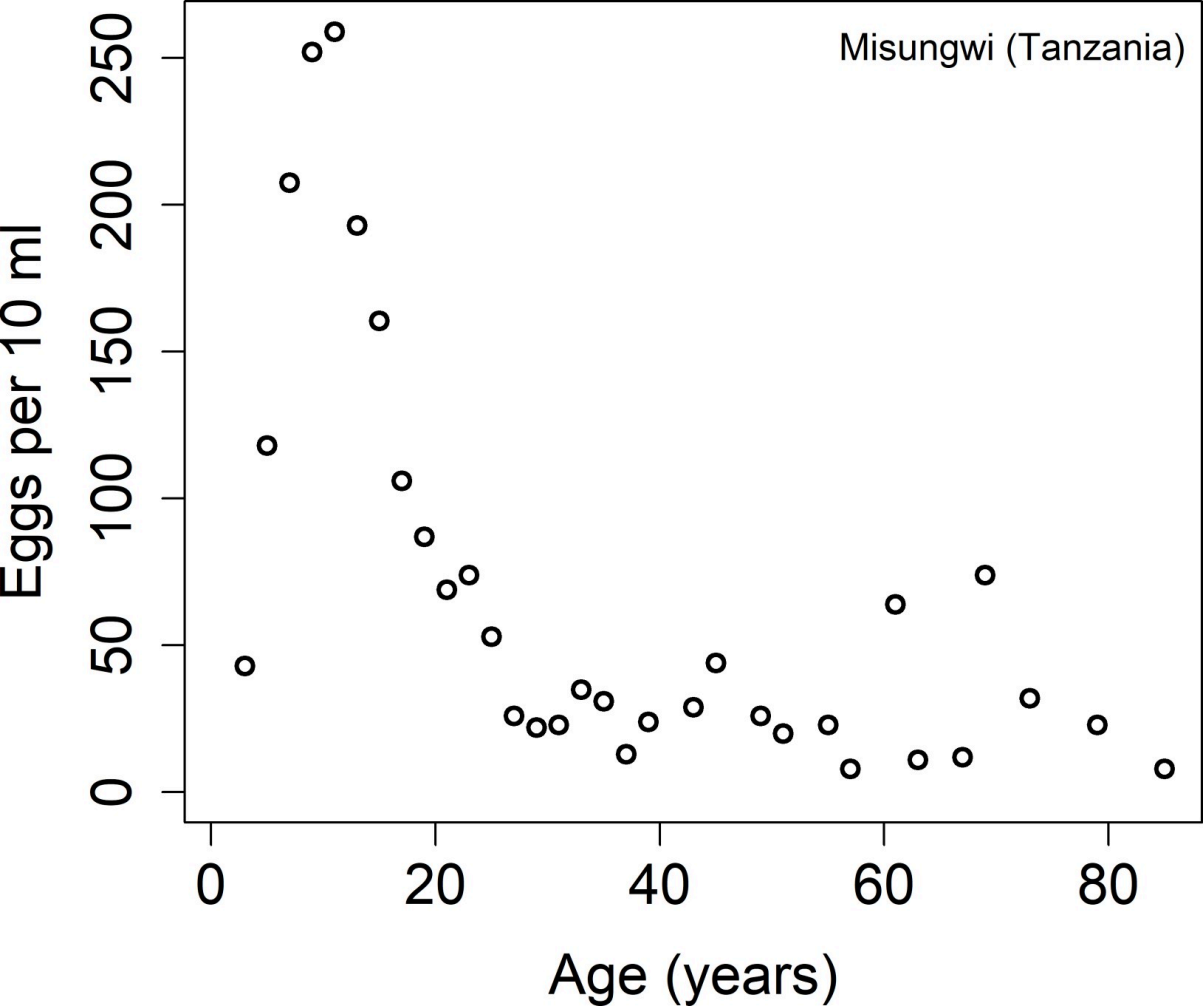
Age intensity cross-sectional profiles for *Schistosoma haematobium* from Tanzania [14].

There are two main reasons for targeting mostly the school age children. The first one is that they are more likely to harbour high levels of infection. In Fig 1 we record the age-intensity cross-sectional profile for *Schistosoma haematobium* from Tanzania [14]. It is clear in this setting that the intensity of the disease increases with age until it reaches the peak in the teens and declines with advancing years. This pattern is typical for all the major schistosome infections in all regions of endemic infection.

Additionally, this SAC age category is easier to be reached by health workers as they can administrate MDA in school settings.

**Fig 2:**
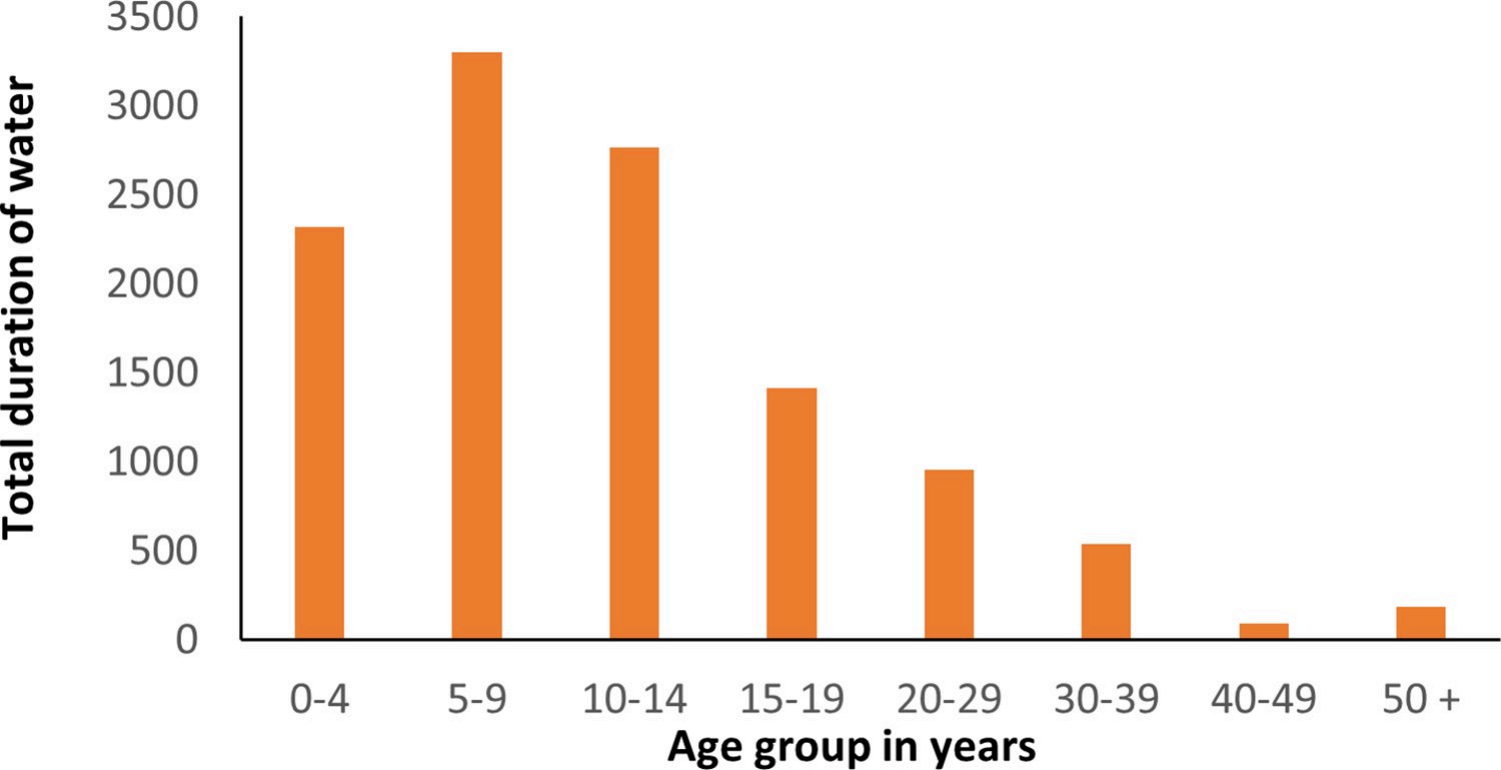
Total duration of water contact (minutes) over an 8-day observation period in St Lucia [18].

Previous studies of transmission and control by MDA have assumed that age-related exposure to infection determine the shape of the age intensity profiles, partly due to the fact that water contact is required for an individual to be exposed to infection (due to the aquatic lifestyle of the snail intermediate host) [3]. There are many published studies which record that the shape of age-related water contact [15–18], is similar to the shape of age-intensity profiles (see Fig 2). This lends support to the argument that ecology/human behaviour plays an important role in determining the shape of the observed age intensity profiles.

Deterministic age structured models of acquired immunity and MDA impact have been developed for human helminth infections back in the 1980s but not applied in a modern context for analyses of the impact of WHO guidelines in the control of NTDs [19,20]. The question of ‘ecology or immunology’ determining the convex shape of age intensity curves of infection is still not resolved [21], but a degree of immunity is believed to slowly build up over long periods of exposure given ample evidence of humoral and cellular immunological responses to schistosome antigens in the blood of infected individuals [19,22]. To date, there is limited information to explain how effective the slow development of acquired immunity is in restricting parasite establishment and growth to reproductive maturation in the human host. Some animal model work on repeated exposure to *S*. *mansoni* in mice suggest it can play a role in generating convex age intensity profiles [23].

Various authors have possible reasons for the slow development of anti-schistosomiasis immunity. (i) One hypothesis is related to the assumed long parasite lifespan (many years). Dying worms (natural death rates) are the main source of antigens that stimulate a strong immune response [24–26]. (ii) A second hypothesis is a certain level of antigen (probably high) is required to stimulate a protective response [27]. Deterministic and individual-based stochastic models have been used to show that it is challenging to distinguish between the two hypothesis [28].

Acquired immunity has been demonstrated in humans and in animal models (by detection of humoral antibodies and cellular reactivity) [21,29,30]. In 1963, a German researcher, used himself as a subject to show the presence of the acquired immunity by exposing himself (on 16 occasions over a period of several months) to cercariae from *Bulinus truncatus* snails. At the end of the experiment he never experienced any sign of symptoms of infection and did not find any eggs in his urine [14].

Various authors have developed simple and complex models (with or without age structure) of the build-up of acquired immunity [15,31,32] by assuming that it acts on different rate parameters such as worm establishment, fecundity or worm survival. Building on past work of [31], Chan et al addressed the role of the acquired immunity in schistosomiasis control and described the key parameters that need to be measured in order to improve the model predictions [33].

Although the age-related exposure to infection alone, or acquired immunity alone, can produce the age-intensity profile for schistosomiasis, it is very likely that convexity in age–intensity profiles is a combination of both mechanisms. In this study we include acquired immunity in our model, building on past work in this area of model construction by Anderson & May [20]. We fit the model to cross-sectional age intensity data for *S*. *haematobium* from Tanzania as described in [14] (Fig 1) and analyse its effect on achieving the WHO goals of morbidity control and elimination as a public health problem.

## Methods

The main hypotheses of this work are as follows (i) Given the context of the original and still unresolved, question originally posed by Warren [21] about what the dominant mechanism behind the shape of age-intensity profiles for schistosome infections might be (immunity or ecology): acquired immunity is hypothesized to have a significant impact on the inferred epidemiological patterns from these profiles when included in the population dynamics for real epidemiological age prevalence and age intensity data sets. (ii) Building from the consequences of confirming (i): the inclusion of both the hypothesized modifications to the inferred epidemiological parameters (age dependent exposure) and acquired immunity in simulated populations will significantly affect the outcomes of MDA impact forecasts for schistosomiasis infections. (iii) Administrating MDA at a rate less than that required to eliminate schistosomiasis can increase mean worm burden in the older age categories above the pre-treatment levels and reduce the level of acquired immunity [19,32].

It should be noted that the epidemiological importance of assessing the above hypotheses is that most inferred age-intensity profiles and MDA impact forecasts based on models of parasite transmission in the literature do not yet include the effects of acquired immunity. Behavior determining the rate of exposure to infection is assumed to be the only mechanism in most recent published studies [3]. The original work on models that include acquired immunity was completed by Anderson & May in the 1980s and published in Nature. However, these early models of the impact of immunity were not fitted to data to infer parameter estimates nor did they consider how the build-up of immunity impacts the design of MDA programmes for parasite control [19].

### The mathematical model of transmission, acquired immunity and control by MDA

In this section we describe an individual-based stochastic model defined in past published research [31,32] based on the parasite transmission framework described in [9] (and also in S1 Text). Human hosts are modelled individually, and the number of worms is tracked for each person over time and in each age class. The (i) acquisition and death of worms within each individual host and (ii) births and deaths of human population, are modelled as distinct events. In Table A in S1 Text we have summarized the stochastic events with their associated rates. Acquired immunity is added into this framework as originally described by Anderson and May [3] as described in the following section.

Assume that immunity is acquired as a result of human host exposure to worm infection, the severity of the response is assumed to be proportional to the cumulative past infection load in an individual of age *a* over the past *a* years [19]. In the model, we assume that acquired immunity acts on fecundity and protects the host against further infection by decreasing the rate at which parasite establishment occurs. We also assume that the strength of acquired immunity is not lifelong, but decays over time. We denote by *θ* the decay rate of acquired immunity, and therefore the average duration of protection is $\frac{1}{\theta }$.

If *M*(*a*,*t*) is the mean worm burden at age *a* and time *t*, then the average level of acquired immunity *I*(*a*,*t*) is as follows:

$$I(a,t)=\int_{0}^{a}exp(-\theta (a-a^{\prime }))M(a^{\prime },t-a+a^{\prime })da\prime$$

Then the rate of change of mean worm burden in the deterministic (mean field) model for the stochastic simulation is:

$$\frac{\partial M(a,t)}{\partial t}+\frac{\partial M(a,t)}{\partial a}=\Lambda (a,t)e^{-\delta I(a,t)}-\sigma M(a,t)$$

An exponential form (as opposed to a linear relationship of direct proportionality) is employed to relate the degree of acquired immunity in terms of the protection offered where *δ* is a measure of the protective strength. Λ(*a*,*t*) = *L*(*t*)*β*(*a*) is the age-dependent force of infection where *L* is the concentration of the infectious material in the environment (infected snails releasing cercaria), *β*(*a*) describes the age-dependent contact rates and *σ* describes the worm death rate [20,31].

Egg counts for individual hosts of age *a*, denoted by E(a), is given by:

$$E(a)=\lambda M(a)f(M(a),z,k)e^{-\delta I(a,t)}$$

where λ is the mean egg per gram output from a worm pair in the absence of density-dependent fecundity effects (see Table 1). The function *f*(*M*(*a*),*z*,*k*) describes the production of fertile infectious material (see S1 Text).

**Table 1:** Parameter values for *Schistosoma haematobium* used in making acquired immunity model predictions of MDA impact (some directly estimated from age-intensity or prevalence profiles and some derived from the literature). The value in square brackets indicate the 95% Credible Intervals.

Parameter	Value	Source
Population size	500	-
Fecundity	3.6 eggs/female/sample	[37,38]
Basic reproduction number	R_0_ = 2.55 [2.1–2.8]	Fitted
Level of aggregation of parasites in host population	Negative binomial, *k* = 0.24	[35]
Adult worm life expectancy	4 years	[20,31]
Praziquantel drug efficacy	99%	[38,39]
Protective strength (***δ***)	0.002	[19,32]
Decay rate of acquired immunity (***θ***)	0.2	[19,32]
Age specific contact rates (***β***).	For 0–4,5–11,12–22,23+ years of age: 0.18 [0.13–0.20],1,0.14 [0.03–0.18], 0.037[0.028–0.043]	Fitted
Contribution to the reservoir by contact age group (***ρ***).	For 0–4,5–11,12–22,23+ years of age: 0.18 [0.13–0.20],1,0.14 [0.03–0.18], 0.037[0.028–0.043]	Fitted
Prevalence of infection	Percentage of population having >0 eggs/10ml	-
Heavy-intensity infection prevalence	Percentage of population having ≥50 eggs/10ml	[1,40]
Human demography	Based on Kenya’s demographic profile	-

This model assumes monogamous sexual reproduction among worms, density dependent fecundity and a negative binomial form for the distribution of parasite numbers per host with a fixed aggregation parameter *k*.

This model has a full age structure for the human host, but outputs are grouped into three age categories, pre-SAC (0–4 years of age), SAC (5–14 years of age) and adults (15+ years of age). We use these age categories based on WHO definitions of treatment groups to calculate the necessary MDA coverage levels for each category in order to control or interrupt transmission.

We assume the time scale of the dynamics of the infective stages (cercaria) is very short (days) in comparison to that of the adult worm in humans (years) so the dynamics of the infective stages can be collapsed into the equations for the mean worm load [9].

Using this model, we can develop a sampling algorithm to infer the age specific contact rates and *R*_0_.

### Data used and parameter estimation

In this study, we use *S*. *haematobium* age-intensity of infection baseline data from a cross-sectional study of 4168 people from the Misungwi area in Tanzania (Fig 1), with no history of MDA prior to data collection. The details of data collection and examination are described in reference [14]. Briefly, for each individual, the average egg-counts of two urine samples of 10 ml were recorded using the urine filtration. Heavy-intensity infection was detected when the number of eggs per 10 ml was larger than 50.

Most of parameter values used in this paper are taken from within ranges found in the literature due to difficulties in their measurement from a single data set. However, data for the age-specific contact rates (*β*), age-specific contribution of hosts to the reservoir (*ρ*) and *R*_0_ are unknown. Using a Bayesian approach, we estimate these by fitting our model to the eggs per 10 ml age profile.

**Fig 3:**
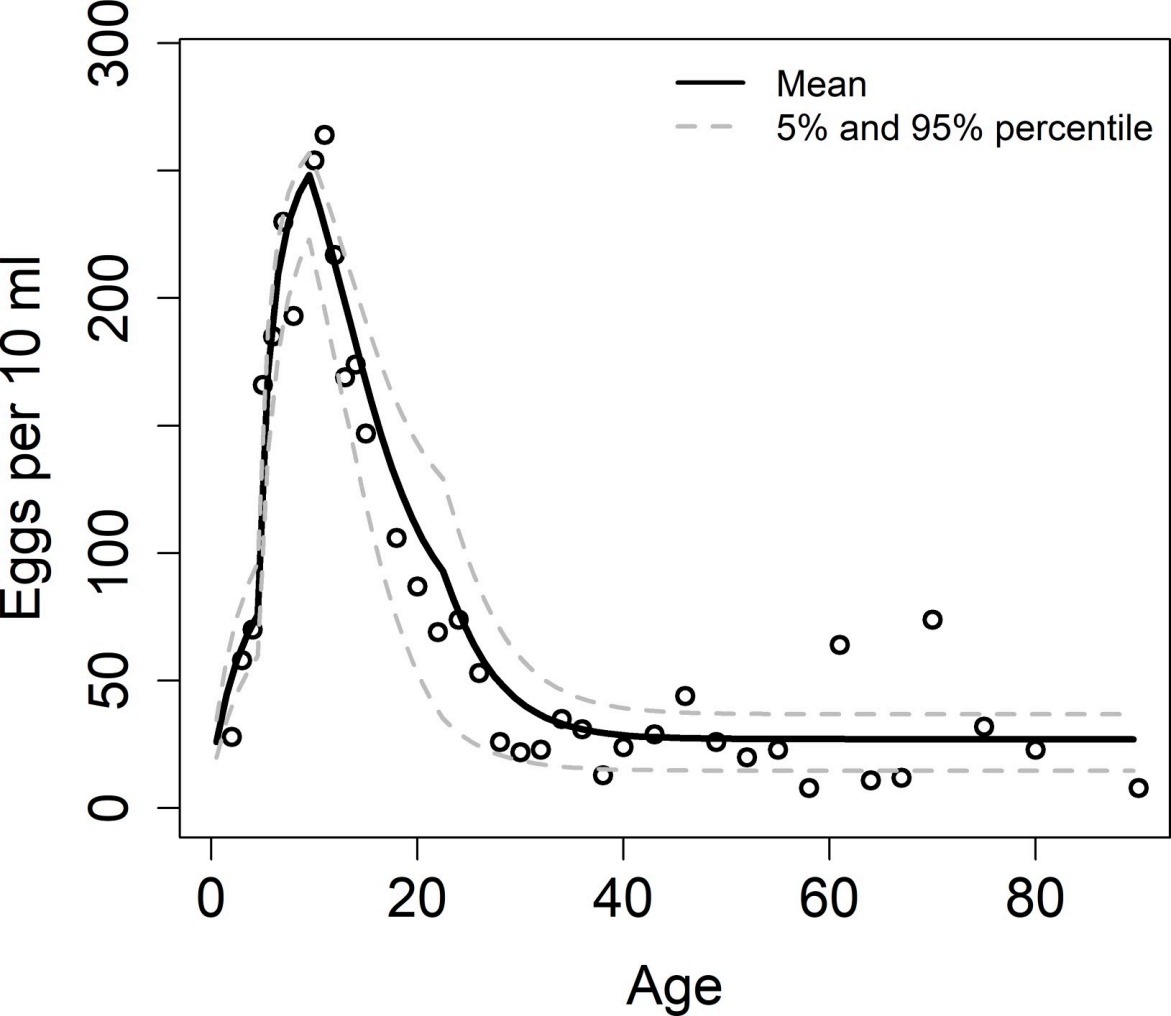
MLE fit as a function of age (in years) to intensity data for *S*.*haematobium* (data from Misungwi area (Tanzania) [14]).

In our model we assume the host contribution to the reservoir to be the same as the age-specific contact rates. Age categories for the contact parameter *β* are 0–4, 5–11, 12–22, 23+ years and are chosen to obtain the best fits to the observed trends. Using Markov Chain Monte Carlo (MCMC) Metropolis-Hastings algorithm (50000 iterations), we estimate *R*_0_ and *β* in the observed age categories (Fig 3), while the values for the acquired immunity are fixed [19,32]. The MCMC chain was constructed using the MCMC package in R (version R-4.0.2). The convergence is assessed by plotting (i) the values of the parameters against the iteration number and (ii) the marginal scatterplots of sampled values from the posterior distribution of fitted parameters (Fig A in S1 Text). The parameter estimation method has been described in details elsewhere [34] and has been used in previous studies [3,35,36]. Table 1 provides a list of all parameters estimated and those taken from within the ranges recorded in the literature.

Host demographic details were taken from published data on sub-Saharan countries with the profile in Kenya chosen to represent a typical profile where S. haematobium is endemic.

Using the acquired immunity model and parameter values defined in Table 1, we simulate low to high transmission settings and followed the current WHO treatment guidelines for MDA to control schistosomiasis (for SAC and adults), as described in the introduction to this paper. The basic reproduction number *R*_0_, is varied in model to simulate this range of baseline prevalence settings (such that the baseline prevalence increases with *R*_0_). In our simulations, we use aggregation parameter *k* = 0.04 value for low transmission setting and *k* = 0.24 for moderate to high transmission settings. In scenarios where we cannot achieve morbidity control and EPHP for up to 15 years of treatment (by following the WHO-recommended treatment guidelines), we propose programmatic adaptations by increasing the coverage levels/treatment frequency and/or including adult treatment.

In this model we assume no migration where the simulations are run for a single community of 500 individuals. We also assume that treatment is delivered at random at each round i.e. no systematic non-adherers/compliers and no non-access to treatment by any individuals in the defined population. To project the expected MDA impact in reducing the intensity of infection, we record the prevalence and the heavy-intensity prevalence of infection in SAC as the average of 300 simulations (to ascertain the mean expectation of the stochastic model) of the stochastic model and record the variability around the average trend across all the individual simulations. For each scenario we calculate the probability of achieving the WHO goal. The goal is achieved when this probability is >0.8, likely to be achieved when the probability is 0.5–0.8 and not achieved when the probability is <0.5.

## Results

To estimate the effect of acquired immunity in morbidity control and elimination, we compare the results with those obtained under the assumption of no immunity, i.e., the age-intensity profile is described by the age-related contact rates only (*R*_0_ = 2.55) [12]. Table 2 records the simulations results for no acquired immunity for *S*. *haematobium*.

**Table 2:** Summary of model projections after following the recommended guidelines set by WHO and suggestions for programmatic adaptations in cases where the WHO goals are not achieved for *S*. *haematobium* (no acquired immunity is assumed) [12].

Baseline prevalence in SAC	Morbidity goal reached?	EPHP goal reached?	Programmatic adaptation
**Low (< 10%)**	Yes, within 3 years	Yes, within 15 years	NA
**Moderate (47%)**	Yes, within 11 years	Not reached	Include adult treatment at 40% coverage
**High (≥ 50%)**	Yes, within 15 years	Not reached	Increase coverage to 85% for SAC and include adult treatment at 40% coverage

*β*_0−4_ = 0.17, *β*_5−11_ = 1, *β*_12−20_ = 0.11, *β*_21+_ = 0.035 are estimated by fitting the model to intensity data for *S*.*haematobium* (data from Misungwi area (Tanzania).

**Fig 4:**
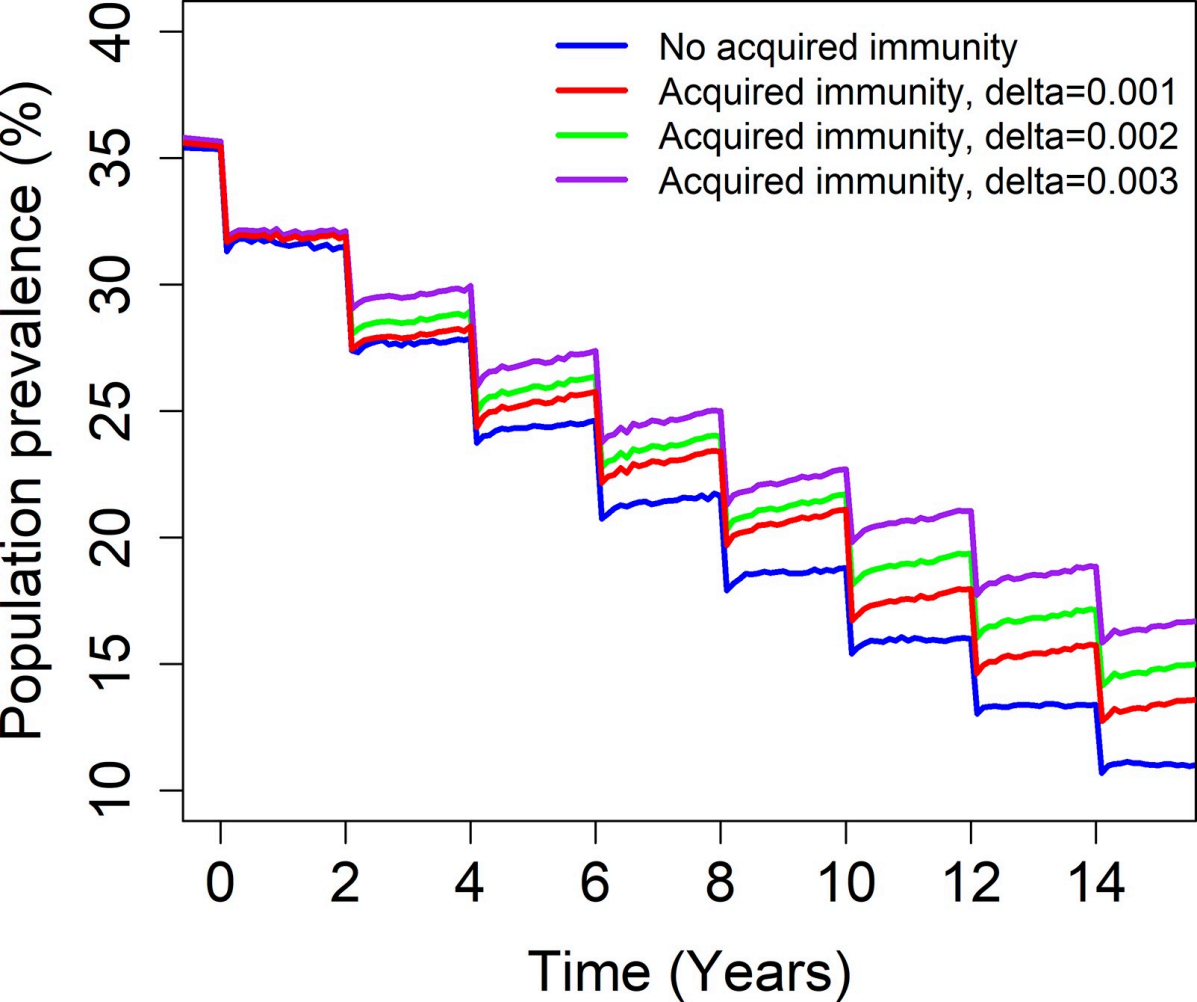
Model projections of MDA treatment of 75% school-aged children (SAC; 5–14 years of age) in moderate baseline transmission settings. The blue line shows the impact of MDA programme assuming that there is no acquired immunity present. The red line shows the impact of MDA programme assuming that there is acquired immunity present with *δ* = 0.001. The green line shows the impact of MDA programme assuming that there is acquired immunity present with *δ* = 0.002. The purple line shows the impact of MDA programme assuming that there is acquired immunity present with *δ* = 0.003. The model was fitted to data for each value of *δ*.

The results from the simulations show that MDA lowers the prevalence of infection in the community regardless of the presence of acquired immunity (Fig 4). However, the impact of MDA is felt more slowly under the influence of acquired immunity for various degree of strength, *δ* (low and medium to high). There is a lesser reduction in the prevalence of infection. This is due to the fact that repeated MDA with a coverage and treatment frequency less than that required to eliminate the disease caused by infection, can reduce the level of acquired immunity over time since the start of MDA and consequently increase average worm burden in adults above the pre-control levels. The change in the prevalence occurs due to lower levels of acquired immunity in the adults age–category as a result of reduced intensity of transmission. Reduced immunity means higher worm burdens. This behaviour will be more evident if strong and lasting immunity is acquired and in areas with a high transmission. This outcome is in line with previous results shown in [19]. This clearly shows that the acquired immunity is an important factor when considering elimination or morbidity control of schistosomiasis.

An associated question is does acquired immunity influence the time to morbidity control and EPHP goals depending on the baseline prevalence in SAC as defined by the WHO (low, moderate and high transmission settings) and treatment frequency (once every three years in low transmission settings, once every two years in moderate transmission settings and once a year in high transmission settings)?

**Fig 5:**
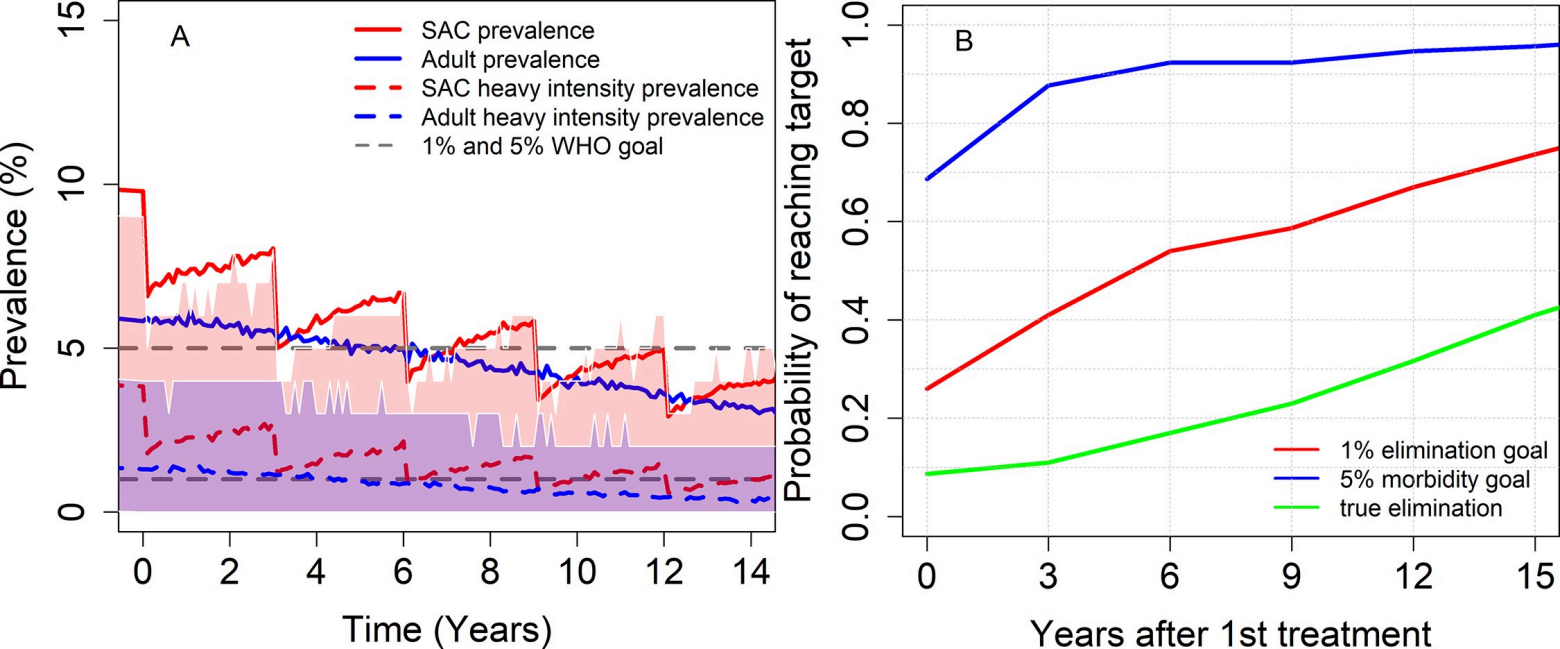
Model projections of MDA treatment of 75% school-aged children (SAC; 5–14 years of age) in low baseline transmission settings. δ = 0.002 is considered. Graph (A) shows the prevalence of infection and heavy intensity prevalence in SAC and Adults over time and graph (B) shows the probability of achieving morbidity control and EPHP. Shaded areas (both blue and red) represent the 90% credible interval (90% of the simulated results fall within these shaded areas) for heavy-intensity prevalence in SAC and Adults. True elimination (interruption of transmission) is achieved when the incidence of new infections in a community is reduced to zero.

For low baseline transmission settings (*R*_0_ = 1.5), we find that the morbidity control goal can be achieved within 3 years of MDA, with a probability >0.8. The EPHP is likely to be achieved with a probability of approximately 0.72 after 15 years of treatment (Fig 5). The acquired immunity reduces the probability of achieving the morbidity control goal, but it does not affect the time to reach this goal as the probability of achieving this is still very high. However, the acquired immunity does affect the time to EPHP, as it will take longer than 15 years to achieve this goal.

Therefore, in low transmission settings the impact of acquired immunity depends on the goal.

For moderate transmission settings (*R*_0_ = 1.8−2), we find that results are dependent on the baseline prevalence prior to MDA treatment (Table 3). For baseline prevalence among SAC less than 40%, it might take up to 10 years to achieve the morbidity control goal. For baseline prevalence among SAC above 40%, the morbidity control goal is not achievable within 15 years of treatment. If acquired immunity was not present (Table 2), treating 75% of SAC can achieve this goal for all moderate baseline transmission settings. For the elimination as a public health problem goal, we find that it is highly unlikely that this goal can be achieved by treating 75% of SAC only once every two years (probability <0.5). To achieve morbidity control and EPHP for baseline settings > 40% we need to increase the SAC coverage/include adults in our treatment at a coverage above 75% for SAC and 40% for adults or increase the treatment frequency.

**Table 3:** Number of years to achieve the goal (with a probability >0.8) for moderate and high transmission settings (acquired immunity is assumed to be present). MDA is administrated for 15 years.

Target goal	Low to Moderate transmission settings	High transmission settings
**Morbidity control**	Baseline SAC prevalence <40%: 2–10 years Baseline SAC prevalence >40%: Not achieved	Baseline SAC prevalence <57%: 11–14 years Baseline SAC prevalence >57%: Not achieved
**EPHP**	Baseline SAC prevalence <15%: 14 years Baseline SAC prevalence > 15%: Not achieved	Not achieved

**Fig 6:**
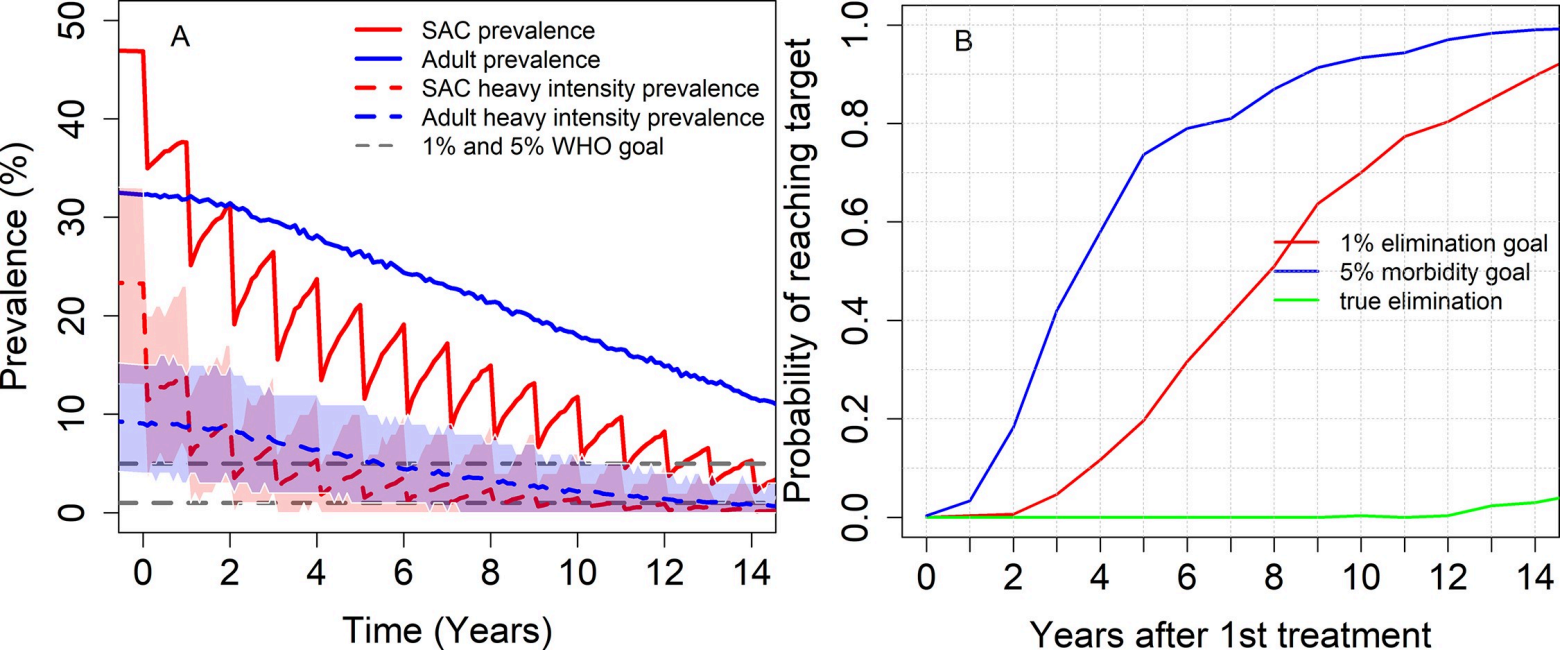
Model projections of annual MDA treatment of 75% school-aged children (SAC; 5–14 years of age) in moderate baseline transmission settings. δ = 0.002 is considered. Graph (A) shows the prevalence of infection and heavy intensity prevalence in SAC and Adults over time and graph (B) shows the probability of achieving morbidity control and EPHP. Shaded areas (both blue and red) represent the 90% credible interval (90% of the simulated results fall within these shaded areas) for heavy-intensity prevalence in SAC and Adults. True elimination (interruption of transmission) is achieved when the incidence of new infections in a community is reduced to zero.

From Fig 6, we see that increasing the treatment frequency to once a year can achieve the morbidity control goal and EPHP (with a probability > 0.8) within 8 and 13 years from the start of MDA, respectively.

**Fig 7:**
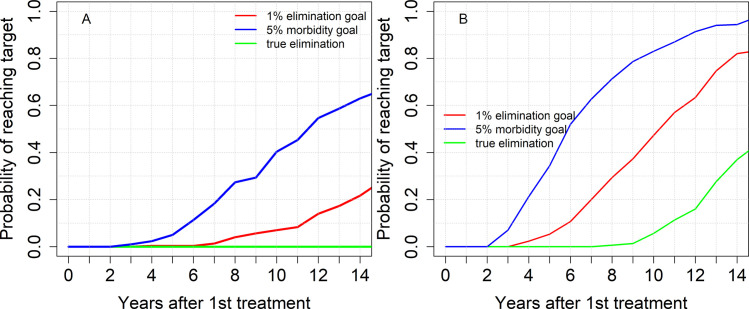
**Model projections of annual MDA treatment of 75% school-aged children (SAC; 5–14 years of age) (Graph A) and 85% SAC + 75% Adults (Graph B) in high baseline transmission settings. δ = 0.002 is considered.** True elimination (interruption of transmission) is achieved when the incidence of new infections in a community is reduced to zero.

For high transmission settings and baseline prevalence less than 57% (*R*_0_ = 2.53), the morbidity control goal can be achieved after many years of MDA (15 years). For baseline prevalence above this threshold, this goal is not achieved under the current WHO guidelines. Additionally, the EPHP is not achieved by treating 75% of SAC only (Table 3 and Fig 7A). Increasing the SAC coverage to 85% and including 75% of adults in our treatment can achieve the morbidity control goal within 10 years and the EPHP within 14 years (Fig 7B). Thus, it will take a long time to achieve control and elimination even with high community-wide coverage. Achieving this high coverage is likely to be very challenging due to various factors such as limited availability of PZQ and evidence of non-compliance to treatment. Hence it is very unlikely that we can achieve these targets for high baseline prevalence (= high pristine transmission settings).

## Discussions

The question of whether humans acquire some level of immunity to schistosome parasite infection post exposure is of great biological interest as well as being important for disease control and elimination by mass drug administration. Acquired immunity if present is a major determinant of infection intensity in individuals in endemic region and has an important effect on the observed epidemiology of schistosome infection in the sense of influencing the shape of age-intensity profiles and the impact of interventions to reduce parasite burdens. [19,22]. If acquired immunity is strong, then parasite control by MDA could in principal impact rates of reinfection once drug treatment ceases. Even though there is evidence of some level of immunity which is acquired gradually over many years of exposure it is thought to provide only partial protection against further infections. Most of the published mathematical modelling assume that the age-intensity profile is largely due to age-dependent exposure to infection not the build-up of immunity. The reason for this is the complete absence of quantitative data available on the duration and strength of acquired immunity acting to reduce the establishment of adult worms in the human host as a function of past exposure. From cross sectional data on the intensity of infection plus water contact observational studies, it is very challenging to distinguish between the relative roles of acquired immunity and age-dependent infection contact rates as originally noted by Warren [21]. Interpretation is made more complex by the high variance in worm loads as judged by egg output within samples of people of similar ages in given transmission settings.

Two of the important WHO 2020 goals for schistosomiasis have been morbidity control and elimination as a public health problem (EPHP). The WHO recently launched NTD control Roadmap for 2021-to 2030 and EPHP is the only goal set for schistosomiasis. Most of the previous modelling work that is done to analyse the criteria needed for these goals to be achieved (in terms of MDA coverage and frequency of application), has not considered the effect of acquired immunity on the intensity of transmission under MDA impact. We extend the schistosomiasis transmission model [15,20], by including acquired immunity and analyse its impact on schistosomiasis morbidity. We assume that immunity is related to the cumulative past infection of an individual and that it protects the human host against further infections by decreasing the rate of parasite establishment in a non-linear manner as defined in the Methods section. The strength of this immunity is assumed to decay over time at a defined rate. The model is fitted to data describing age intensity of infection profiles for *S*. *haematobium* as described in [14]. The model assumptions and some parameter values are based on previous studies [19,22,31,32].

To explore the impact of acquired immunity on MDA programmes, we compare results with those obtained under the assumption of no immunity, but with age-dependent exposure.

Our model-based analysis demonstrates a slower decrease of the intensity of infection when acquired immunity is present than when it is absent, meaning that MDA programmes are less effective. The exact impact that it has on intensity of disease depends on the baseline prevalence prior to treatment (the magnitude of the basic reproductive number R_0_), coverage levels and treatment frequency. In low transmission settings, we find that even though the prevalence of infection is slowly decreasing when acquired immunity is present, it does not affect the time to achieve the morbidity control goal target (compared with the no-immunity model) as it is achieved early into the programme. On the other hand, acquired immunity increases the time to achieve EPHP compared to when it would have been achieved if no immunity were present (Table 2). We also show that increasing the strength of immunity build up by altering the parameter defining the strength of immunity related to cumulative past exposure reduces the impact of MDA since the action of MDA on children to lower infection acts to reduce the build-up of immunity in later life as they age (Fig 4). This means that when MDA includes children, who have not experienced as much past exposure and hence immunity maturation, this lowers their immune response to potential exposures in later life when MDA might not be administered so frequently or indeed not at all in MDA programmes only targeted at children. Hence, the impact of MDA for children in later life may not necessarily be as positive as it might initially be anticipated if it prevents the build-up of a degree of acquired immunity protection.

In moderate transmission settings, the results depend on the baseline prevalence (the magnitude of R_0_) and the goal. Morbidity control goal can be achieved for prevalence threshold less than 40%, while the EPHP can be achieved for prevalence threshold less than 15%. To increase the probability of achieving these goals, higher treatment frequency or community-wide MDA is needed. This outcome is in line with previous results presented in [41], which reported that increasing SAC coverage levels can give a greater reduction in egg output. If immunity was not present, we could achieve the morbidity control goal (for all baseline transmission settings, being low, moderate and high) as defined in the current WHO guidelines, but the treatment programme might need to be adapted for the EPHP goal (Table 2).

Similar results are obtained for high transmission settings. We find that it is very unlikely that the morbidity control goal can be achieved within a short period of time. In comparison with no immunity scenarios, the morbidity goal could be achieved within 15 years of treatment. We also find that the EPHP goal is not achieved under the current treatment guidelines. Increasing the protective strength (*δ*), increases the number of years to achieve the EPHP goal (Fig B in S1 Text).

It is apparent from our modelling results that acquired immunity, if significant, has a considerable impact on schistosomiasis transmission and its control and elimination by MDA and other interventions that reduce to some degree exposure to infection but not entirely eliminate exposure. We show that the MDA programme might be less effective if some form of immunity is acting. This means that previous modelling results might be on the optimistic side when it comes to achieving morbidity control and the WHO elimination as a public health problem goal. We have no estimates of the possible severity of the build-up of immunity hence we explored three different scenarios (low, moderate and strong build up as a function of accumulated past exposure). The stronger the impact the more likely past work has been too optimistic on MDA impact to achieve the WHO goals.

We also assumed that treatment is delivered at random at each round i.e. no systematic non-adherers and no non-access to treatment individuals. This is likely not the case, as non-adherers are a feature of most MDA programmes [42]. Recent work on MDA to control soil transmitted helminths has revealed quantitative evidence on non-adherence [43,44]. If non-adherers are not specifically targeted by health workers over a many years MDA programme, then our results will be on the optimistic side. Systematic non-compliance to treatment will make it more challenging to achieve morbidity control and elimination, particularly in moderate to hight transmission settings. This will require an adaptation of the current treatment guidelines, to increase treatment frequency or including adults in the treatment programme.

We acknowledge, that due to limited PZQ availability, this might not be feasible for all settings. Hence it is important that SAC and adult epidemiological data on the intensity and prevalence of infection are collected prior to initiation of MDA programmes to assess the degree of prevailing transmission, so that the optimal treatment strategy is applied by health workers in terms of treatment frequency in high transmission settings.

In this work, we have assumed that adult worm life expectancy is 4 years. However, the effect of MDA in controlling transmission is sensitive to this parameter. Previous work by Mitchell et al has showed a lesser reduction in egg output for shorter worm life span [41].

Other assumptions will affect the accuracy of the model predictions. Parameter assignments are well supported by data in some areas, but in others, especially the nature of acquired immunity and its relation to past exposure, information is absent at present. We assumed in the absence of hard data that the acquired host response reduced the parasite establishment rate and fecundity. However, it could also act to reduce adult worm survival and adult worm growth rates [20]. We also assume that the immunity decays exponentially since the exact form of the decay function is also not known. Again, in the absence of firm epidemiological data this may be either a pessimistic or optimistic assumption. The question originally posed by Warren of ecology or immunity or both still remains unresolved [21].

## Supporting information

S1 Text**Fig A:** Marginal scatterplots of sampled values from the posterior distribution of fitted parameters. **Fig B:** Number of rounds to achieve EPHP for various combinations of the protective strength (*δ*) and duration (years; $\frac{1}{\theta }$) of acquired immunity (moderate transmission setting). **Fig C:** Predicted time series for mean worm burden in the population and for MDA treatment of 75% school-aged children (SAC; 5–14 years of age) in low baseline transmission settings. **Table A:** Table of events for the stochastic model (as in [3]), where *λ*_*i*_ is the gamma distribution for individual *i*, *δ*() is the Dirac delta function, *g* is the proportion treated, *N*_*i*_ is a host’s total worm burden (of which *n*_*i*_ are female worms) and Ber() is a Bernoulli-distributed random variable.(DOCX)

S1 Data Simulation outputs. (XLSX)
