# Supplementary material for: What is the impact of acquired immunity on the transmission of schistosomiasis and the efficacy of current and planned mass drug administration programmes?
Source: PLoS Negl Trop Dis. 2021 Dec 1;15(12):e0009946. doi: 10.1371/journal.pntd.0009946 (PMC8635407; doi:10.1371/journal.pntd.0009946)
Supplement: S1 Text — Fig A: Marginal scatterplots of sampled values from the posterior distribution of fitted parameters. Fig B: Number of rounds to achieve EPHP for various combinations of the protective strength (δ) and duration (years; 1θ) of acquired immunity (moderate transmission setting). Fig C: Predicted time series for mean worm burden in the population and for MDA treatment of 75% school-aged children (SAC; 5–14 years of age) in low baseline transmission settings. Table A: Table of events for the stochastic model (as in [3]), where λi is the gamma distribution for individual i, δ() is the Dirac delta function, g is the proportion treated, Ni is a host’s total worm burden (of which ni are female worms) and Ber() is a Bernoulli-distributed random variable. (DOCX) [file pntd.0009946.s001.docx]

**S1 Text. Supplementary Information**

**The Model (without acquired immunity)**

The fundamental model used to describe the mean worm burden of individuals of a given age and the quantity of infectious eggs in the environment was developed from the founding work of Anderson and May [1]. The current version of the model is described in detail in [2, 3]. Briefly, the model is an PDE model describing the evolution of mean female worm burden as a function of age, M(a,t)

$\frac{\partial M(a,t)}{\partial t}+\frac{\partial M(a,t)}{\partial a}=L\beta(a)-\sigma M(a,t)$

where L represents the concentration of the infectious material in the environment, namely, how each individual of age a, contributes to the pool of released eggs. The model describes the evolution of the female worm burden and assumes they are distributed according to an underlying negative binomial distribution with a fixed value of the aggregation parameter k. The dynamics of infectious material is governed by

$\frac{dL}{dt}=\psi\lambda\int_{a=0}^{\infty} M(a)f(M(a),z,k)\rho(a)P(a)da-\mu_{2}L$

Where *ψ* characterises the flow of infectious material into the environment, $\rho\left( a \right)$represents the age-specific relative contribution of infectious stages to the environmental reservoir and *P(a)* is the normalised age distribution for the population. The function $f(.)$ describes the production of fertile infectious material and is the product of a term representing the dampening effect of density dependent fecundity at higher worm burdens [first term] and the catalytic effect of the presence of male worms on sexual reproduction at very low worm burdens [second term]

$f(M(a),z,k)=[1+(1-z)M(a)/k]^{-(k+1)}\phi(M(a),k)$

where $z=e^{-\gamma}$ is the strength of density-dependent fecundity. The function φ approximates the effect of monogamous sexual preproduction (dependant on male-female worm pairs) on egg production [4], where

$\phi(M,k)=1-\frac{(1-\alpha)^{1+k}}{2\pi}\int_{0}^{2\pi} \frac{(1-\cos(\theta))d\theta}{(1-\alpha\cos(\theta))^{1+k}}$

where $\alpha=M/(k+M)$. The parameter *Ψ* and the absolute magnitude of β and ρ are subsumed into the definition of the basic reproduction number, R_0_, that measures the intensity of the transmission cycle.

$R_{0}=\frac{z\lambda\psi}{\mu_{2}}\int_{a=0}^{\infty} \rho(a)P(a)\int_{x=0}^{a} \beta(x)e^{-\sigma(a-x)}dxda$

Assuming a 1:1 sex ratio in worms, the total worm burden is given by $2M(a,t)$ . Egg counts for individual hosts of age a, *E(a)*, can be seen as a component of the contribution of host egg output into the environment and is given by

$E(a)=\lambda M(a)f(M(a),z,k)$

where λ is the mean egg per gram output from a worm pair in the absence of density-dependent fecundity effects (see Table A).

**Model code**

The Imperial College London deterministic model code (for no immunity model) has been made available by the NTD Modelling Consortium (<https://www.ntdmodelling.org/diseases/schistosomiasis-mansoni>).

**Table A: Table of events for the stochastic model (as in [3]), where** $\boldsymbol{\lambda}_{\boldsymbol{i}}$ **is the gamma distribution for individual** $\boldsymbol{i}$**,** $\boldsymbol{\delta()}$**is the Dirac delta function,** $\boldsymbol{g}$ **is the proportion treated,** $\boldsymbol{N}_{\boldsymbol{i}}$ **is a host’s total worm burden (of which** $\boldsymbol{n}_{\boldsymbol{i}}$ **are female worms) and Ber() is a Bernoulli-distributed random variable.**

| Event | Definition | Rate |
| --- | --- | --- |
| Per capita worm acquisition by host $i$ , aged $a$ , per unit of time | $N_{i}\to N_{i}+1$  $n_{i}\to n_{i}+Ber(0.5)$ | $\beta(a_{i})\lambda_{i}L$ per host per unit of time |
| Worm death in host $i$ | $N_{i}\to N_{i}-1$  $n_{i}\to n_{i}-Ber(0.5)$ | $\sigma$ per worm per unit of time |
| Host birth/death for host aged $a$ years | At death, host is replaced with a newborn; $N_{i}=n_{i}=0$ | $\mu\left( a_{i} \right)$ per unit of time |
| Treatment of host $i$, aged $a$ years | $N_{i}=N_{i}*(1-[$MDA efficacy]) | $\delta\left( t-t_{j} \right)g(a_{i})$ |


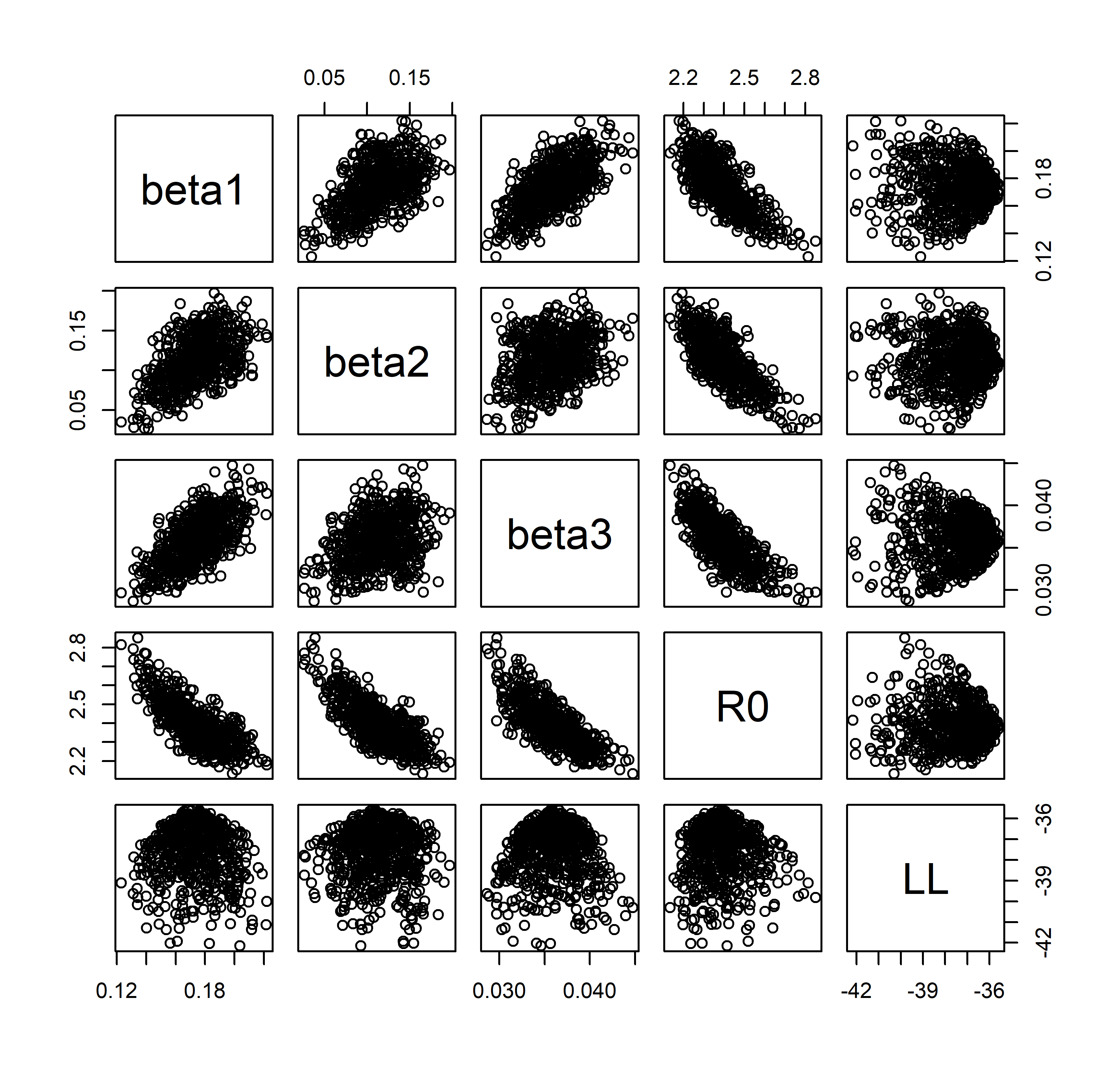


**Fig A.** Marginal scatterplots of sampled values from the posterior distribution of fitted parameters. The beta value (age-dependent relative exposure and contribution to the reservoir) for the 5–11 age group is set to 1. Beta1 is for the 0-4 age group, beta2 is for the 12-22 age group and beta 3 is for the 23+ age group.


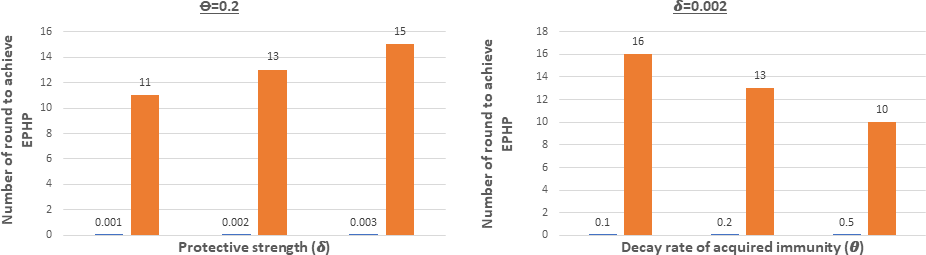


**Fig B.** Number of rounds to achieve EPHP for various combinations of the protective strength ($\delta$) and duration (years; $\frac{1}{\theta}$) of acquired immunity (moderate transmission setting).


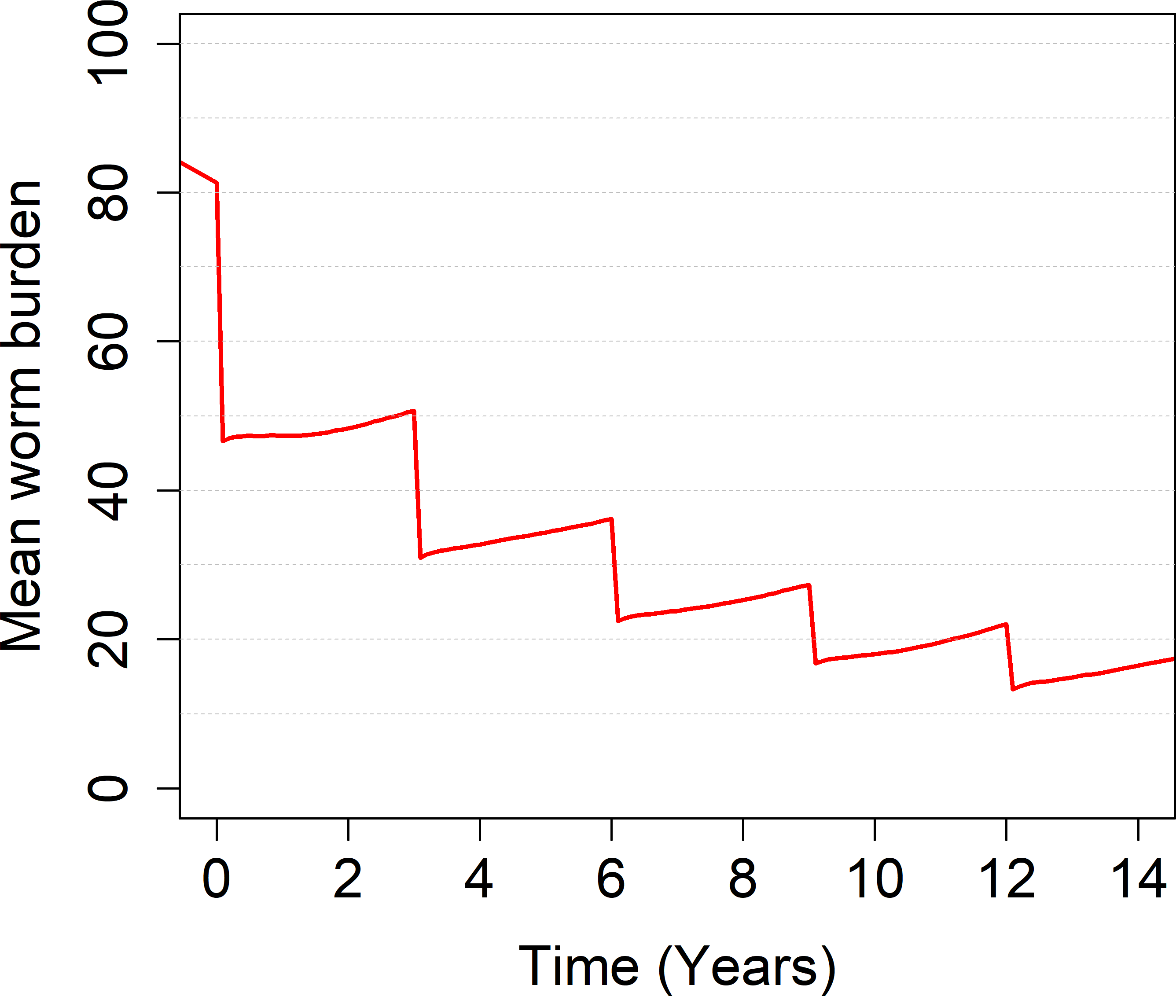


**Fig C.** Predicted time series for mean worm burden in the population and for MDA treatment of 75% school-aged children (SAC; 5–14 years of age) in low baseline transmission settings.

**References**

1. Anderson RM, May RM: Infectious diseases of humans: dynamics and control. Oxford; New York: Oxford University Press; 1991
2. Truscott JE, Hollingsworth TD, Brooker SJ, Anderson RM: Can chemotherapy alone eliminate the transmission of soil transmitted helminths? *Parasites & vectors* 2014, 7(1):266.
3. Truscott JE, Turner HC, Farrell SH, Anderson RM: Soil Transmitted Helminths: mathematical models of transmission, the impact of mass drug administration and transmission elimination criteria. In: *Mathematical Models for Neglected Tropical Diseases: Essential Tools for Control and Elimination. Volume B*, edn. Edited by Anderson RM, Basanez MG; 2015.
4. May RM: Togetherness among Schistosomes: its effects on the dynamics of the infection. *Mathematical biosciences* 1977, 35:301-343.
